# The Perspectives of Combining Antibiotics with Saponins—Herbal Excipients

**DOI:** 10.3390/molecules30204102

**Published:** 2025-10-15

**Authors:** Aleksandra Makiej, Wojciech Smułek, Ewa Kaczorek

**Affiliations:** Institute of Chemical Technology and Engineering, Faculty of Chemical Technology, Poznan University of Technology, Berdychowo 4, PL-60965 Poznan, Poland; aleksandra.makiej@doctorate.put.poznan.pl (A.M.); ewa.kaczorek@put.poznan.pl (E.K.)

**Keywords:** Plant-Derived Bioenhancers, saponins, Sustainable Antimicrobial Strategies, Antibiotic Resistance Mitigation, Human Environmental Health, Eco-Friendly Therapeutic Excipients

## Abstract

The overuse of antibiotics in human and veterinary medicine has contributed significantly to their persistent presence in the environment, creating conditions that promote the spread of antimicrobial resistance (AMR). In this context, innovative approaches that reduce antibiotic doses without compromising therapeutic efficacy are urgently needed. This review explores the emerging role of plant-derived secondary metabolites, particularly saponins, as bioactive excipients capable of enhancing antibiotic activity through synergistic mechanisms. By improving membrane permeability, inhibiting resistance pathways, and modulating host responses, these natural adjuvants may allow for lower antibiotic concentrations in clinical treatments, ultimately reducing pharmaceutical residues entering the environment. We discuss the potential of such combined therapies not only to mitigate the evolution and dissemination of AMR in natural microbial communities but also to provide more sustainable, biodegradable, and ecologically safer alternatives to synthetic formulation agents. Plant-derived compounds, inherently shaped by co-evolution with microbes, offer a dynamic and adaptive molecular diversity that may be less prone to long-term microbial resistance. In addition to reviewing current knowledge, this article highlights the environmental and public health implications of integrating phytochemical excipients into antibiotic regimens, and calls for further interdisciplinary efforts to evaluate their safety, efficacy, and role in shaping future antimicrobial stewardship.

## 1. Introduction

The advent of antibiotics in the last century significantly reduced mortality and morbidity associated with infectious diseases. However, the misuse and overuse of these drugs have precipitated the emergence of resistant microbial strains. Pathogenic bacteria have developed intrinsic resistance to antibiotics through mechanisms such as the alteration of target sites, active drug efflux, and enzymatic degradation [1]. This growing issue has sparked increased interest in medicinal plants, as 25–50% of current pharmaceuticals are derived from plant sources [2]. Crude extracts from these plants represent a promising alternative for resistance-modifying agents due to their diverse array of secondary metabolites. Compounds such as saponins in these extracts possess potential antimicrobial properties and can act as resistance modifiers [3]. Consequently, medicinal plants may also serve as effective modulators of host-related cellular processes, including immune response, mitosis, apoptosis, and signal transduction. Thus, their activity extends beyond merely eliminating microorganisms, as they may interfere with critical stages of the pathogenic process. This multifaceted approach may reduce the ability of bacteria to develop resistance to botanical agents.

This narrative, mechanism-driven review sets out to articulate a membrane-centric rationale for combining licensed antibiotics with herbal bioenhancers, with saponins presented as a translational exemplar. The novelty of this review lies in integrating membrane biophysics with the pharmacology of botanical adjuvants and mapping these mechanisms onto clinically used antibiotic classes and delivery strategies.

## 2. Why We Need New Antimicrobials

**Figure 1:**
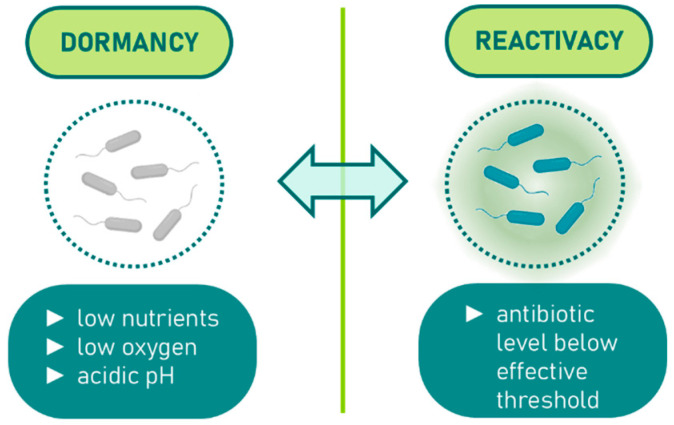
Dormancy induction in bacteria under environmental stress.

The bulk of antibiotic classes in use today were identified during the “golden era” of antibiotic discovery, which occurred between the 1940s and 1980s [1]. During this period, screening efforts focused on compounds capable of inhibiting or killing rapidly dividing bacteria. Consequently, most antibiotics from this era are designed to interfere with crucial bacterial growth processes, such as the synthesis of proteins, peptidoglycan, folic acid, DNA, and RNA [4]. As bacterial resistance to these antibiotics emerged, chemical modifications were applied to existing drug classes to develop analogues with improved potency. These analogues, much like their predecessors, primarily target metabolically active, fast-replicating bacteria. However, in certain infections, bacteria may encounter hostile environments that push them into a quiescent state characterized by minimal or no growth. These metabolically dormant bacteria are capable of surviving high antibiotic concentrations, necessitating prolonged treatment for efficacy [5,6,7,8,9]. Once antibiotic levels drop below the threshold required to kill or inhibit these cells, the dormant bacteria can reactivate, leading to a recurrence of symptoms. Provided that these reactivated bacteria have not developed genetic resistance, subsequent treatment cycles can eliminate them. The shift from active division to dormancy in bacterial cells is complex and often triggered by environmental stresses, such as nutrient and oxygen shortages, and acidic pH, which collectively slow down or halt bacterial growth (as shown in Figure 1).

Infections associated with biofilms and tuberculosis (TB) present significant treatment challenges due to the presence of slow-growing or dormant bacteria. Since the mid-1990s, awareness of biofilm-associated diseases has significantly increased, with the US CDC (United States Centers for Disease Control) estimating that biofilms contribute to up to 65% of all infections in developed countries [10]. These infections range from those linked to medical device implants, such as bloodline catheters and heart valves, to conditions related to cystic fibrosis, wounds, and superficial skin infections. The critical need for improved TB therapies also became apparent in the mid-1990s, particularly with the rise in drug-resistant strains and the deadly interaction between TB and HIV [11,12]. TB remains one of the leading causes of death from bacterial infections, resulting in over 1 million deaths annually [13]. It is estimated that approximately one-third of the global population is infected with dormant, asymptomatic *Mycobacterium tuberculosis*, from which around 8 million new active TB cases arise each year [14].

Over the past decade, extensive research has highlighted the potential of antimicrobials that disrupt bacterial membrane integrity as a novel approach to treating persistent infections. Additionally, bacteria capable of dormancy, such as *M. tuberculosis* and biofilm-forming species like *Staphylococcus aureus* and *Pseudomonas aeruginosa*, may utilize anaerobic metabolism (e.g., substrate-level phosphorylation and anaerobic respiration) to survive without active growth [15,16,17,18,19,20]. Consequently, enzymes involved in these anaerobic processes are being explored as promising targets for new drug development [20,21].

**Figure 2:**
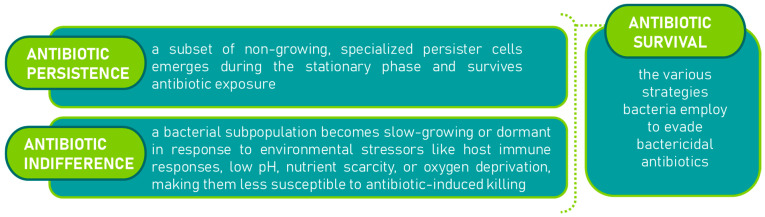
Mechanisms of antibiotic survival [31,32]: persistence [27,28] and indifference [29,30].

Antibiotic resistance is a significant contributor to treatment failures [22]. Beyond genetic resistance, pathogenic bacteria often undergo physiological changes that slow or halt their growth, making them difficult to eradicate with bactericidal antibiotics, despite not having developed genetic resistance [23]. These bacteria can regain antibiotic sensitivity once growth resumes. This recalcitrance to treatment arises from two key phenomena: ‘antibiotic persistence’ and ‘antibiotic indifference’ [24,25,26]. Together, these phenomena, are referred to as ‘antibiotic survival,’ and have been illustrated in Figure 2. Antibiotic survival (as used here) denotes non-inherited phenotypes that allow bacteria to avoid killing by bactericidal antibiotics during exposure and later regain susceptibility upon resumption of growth. We highlight two major states: persistence, a specialized non-growing subpopulation that survives lethal exposure [27,28], and indifference, a stress-imposed slow-growth/dormant state (e.g., host responses, low pH, nutrient or oxygen limitation) that reduces antibiotic-induced killing without genetic resistance [29,30]. As framed by Hurdle et al. and O’Neill [31,32], these non-genetic states are key contributors to treatment recalcitrance.

Emerging consensus from various studies suggests that persisting bacteria evade antibiotic action by significantly downregulating biosynthetic processes targeted by most antibiotics, without compromising their survival in a metabolically inactive state. For instance, β-lactam antibiotics rely on active peptidoglycan synthesis during cell division to activate autolysins [33].

## 3. Targeting the Cell Membrane

Membrane-active agents offer promising antimicrobial properties, making them valuable candidates for drug discovery and therapeutic application. The bacterial membrane is an essential target due to its critical role in maintaining selective permeability, cellular homeostasis, and energy transduction, regardless of the cell’s metabolic state [34,35]. The membrane is composed of approximately one-third of the cell’s proteins and is the site of vital processes, including nutrient and waste transport, bacterial respiration, proton motive force establishment in conjunction with respiratory enzymes, ATP production, and cell–cell communication within biofilms [36]. The antibacterial potential of the membrane is further supported by the action of host-derived antimicrobial peptides and other bioactive molecules that target this structure [37]. Despite the membrane’s importance as an antibacterial target, traditional antibiotic discovery efforts have largely overlooked it. Several synthetic (e.g., ceragenins [38]) and natural compounds (e.g., polymyxins [39]) that damage bacterial membranes, potentially effective against dormant bacteria, have been underexplored, possibly due to concerns about their potential to disrupt mammalian cell membranes and the limited knowledge on optimizing these compounds for pathogen selectivity [40,41].

**Figure 3:**
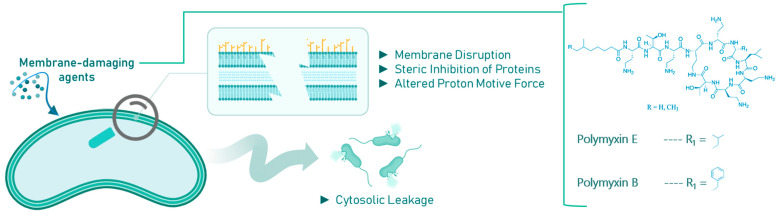
Effects of membrane-damaging agents (with example compounds) on quiescent bacteria.

Membrane-damaging agents often exhibit a complex mode of action, targeting multiple cellular components. These agents can disrupt membrane architecture and functional integrity through the interaction of their lipophilic moieties with the bacterial membrane, sterically inhibit membrane-embedded proteins, or alter the proton motive force, potentially leading to cytosolic content leakage and cell death [42,43,44]. These disruptions can have lethal, pleiotropic effects on quiescent bacteria, as shown in Figure 3.

However, it is important to note that while dissipation of the proton motive force alone is not universally bactericidal, it can limit energy supply in already metabolically inactive cells. Notably, *Mycobacterium tuberculosis* differs from other pathogens in its requirement for a fully energized membrane for survival under both aerobic and hypoxic conditions. As a result, ionophores like nigericin or valinomycin, which dissipate the proton motive force, are highly bactericidal to both active and dormant *M. tuberculosis*. Furthermore, increasing the proton permeability of the membrane enhances the sensitivity of mycobacteria to reactive free radicals, such as nitric oxide and superoxide, within macrophages, a mechanism that is particularly effective at acidic pH levels [45].

## 4. Outer Membrane Permeability and Antibiotic Resistance

Currently, most antibiotics are designed to target intracellular processes, necessitating their ability to penetrate the bacterial cell envelope. The outer membrane of Gram-negative bacteria presents a significant challenge as a robust barrier that antibiotics must overcome [46,47]. Antibiotics penetrate this outer membrane via two primary routes: a lipid-mediated pathway for hydrophobic drugs and diffusion through porins for hydrophilic drugs [48]. The composition of lipids and proteins within the outer membrane significantly influences bacterial susceptibility to antibiotics, and modifications to these components are a common mechanism of drug resistance [49].

The outer membrane (OM) of Gram-negative bacteria serves the crucial function of adding an extra protective layer while still allowing the exchange of materials necessary for life. This dual function highlights the OM as a complex macromolecular structure whose intricacies have only recently been understood. The OM combines a highly hydrophobic lipid bilayer with pore-forming proteins that have specific size-exclusion properties, making it a selective barrier. This selective permeability has a profound effect on a bacterium’s vulnerability to antibiotics, which are generally aimed at intracellular targets [50]. Small hydrophilic drugs, such as β-lactams, use these porins to access the cell interior, while hydrophobic drugs, like macrolides, pass through the lipid bilayer. The emergence of drug-resistant strains across numerous bacterial species, due to alterations in the OM’s lipid or protein composition, underscores the OM’s critical role in antibiotic sensitivity. This review will detail the properties of the OM’s lipid barrier and porin-mediated permeability and discuss how modifications to these structures contribute to antibiotic resistance.

**Figure 4:**
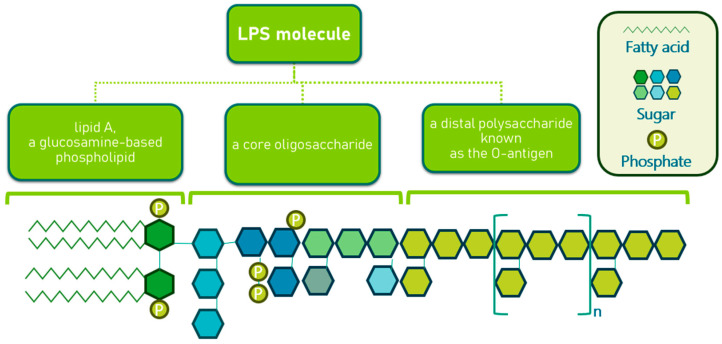
Structure of lipopolysaccharides in Gram-negative bacteria.

In most Gram-negative bacteria, the OM is an asymmetric bilayer composed of phospholipids and lipopolysaccharides (LPS), the latter being exclusively located in the outer leaflet. A typical LPS molecule consists of three components as shown in Figure 4 [51,52]. The inner leaflet of the OM closely resembles the cytoplasmic membrane, containing about 80% phosphatidylethanolamine, 15% phosphatidylglycerol, and 5% cardiolipin [53]. In mutants with altered LPS structures, phospholipids may also be found in the outer leaflet, likely due to a reduction in OM protein levels. The OM is populated by various proteins, some of which are extremely abundant. For instance, murein lipoprotein (Lpp) and outer membrane protein A (OmpA) are present at around 100,000 copies per cell [54]. Lpp is anchored in the OM by a fatty acid moiety, and a portion of Lpp is also covalently attached to the peptidoglycan layer, suggesting a role in maintaining OM integrity [55]. Mutants lacking Lpp produce OM vesicles and lose periplasmic enzymes. OmpA, another abundant protein, is believed to contribute to cell shape, and its absence, along with Lpp, compromises cell structure. OmpA also has pore-forming properties, though with low permeation efficiency, and recent evidence suggests it may exist in two conformations: a monomeric β-barrel and an oligomeric form with large open β-barrels, similar to general diffusion porins like OmpF [55]. Beyond general diffusion porins, the OM includes specialized channels and receptors for the uptake of specific substrates, proteins involved in OM biogenesis, and enzymes such as the OmpT protease in *E. coli* [56]. These components are crucial for various cellular functions, including LPS assembly and the formation of surface appendages like pili and flagella.

Hydrophobic antibiotics, including aminoglycosides, macrolides, and cationic peptides, can permeate the OM lipid bilayer, with the core region of LPS playing a crucial role in providing a barrier [49]. The anionic groups in the LPS structure bind divalent cations, stabilizing the membrane by compensating for electrostatic repulsion between LPS molecules. The lipid-mediated pathway is particularly significant for the resistance of Gram-negative bacteria to hydrophobic antibiotics [49]. Strains expressing full-length LPS exhibit intrinsic resistance to these antibiotics. However, the application of membrane permeabilizers, such as Tris/EDTA or polymyxin B, can dramatically increase the sensitivity of bacteria like *E. coli* and *Salmonella typhimurium* to hydrophobic antibiotics, bringing their sensitivity levels closer to those of deep rough mutants, which have truncated LPS and are more susceptible to lipophilic compounds [46].

Polymyxin B and its nonapeptide derivative, PMBN, disrupt the LPS layer by competing with divalent cations for binding sites, leading to increased lateral diffusion of LPS and destabilization of the OM [57]. This process facilitates the penetration of polymyxin B into the periplasm, where it further permeabilizes the inner membrane, exerting its antibacterial effect. PMBN, though less bactericidal, still enhances OM permeability, demonstrating its potential as an adjuvant in antibiotic therapy.

Targeting the membrane represents a promising strategy for addressing dormant infections, but the discovery and evaluation of membrane-active agents come with several significant challenges and opportunities that need careful consideration. Below, Table 1 has gathered a summary of these key points.

## 5. Antimicrobial Effects of Plant Secondary Metabolites (PSMs)

Historically, plants have been used as medicinal remedies for various ailments. The rising challenge of antibiotic-resistant microorganisms has steered researchers towards plants in search of new antimicrobial agents. Numerous plants produce secondary metabolites as a defense mechanism against microbial and pest attacks, with examples gathered in Table 2.

The structural and chemical complexity of natural products significantly distinguishes them from synthetic drugs. Natural compounds generally contain less nitrogen, sulfur, phosphorus, and halogens, but they exhibit greater molecular diversity, including varied ring systems, carbohydrate content, and stereochemical configurations. These unique properties allow plant products to modulate protein–protein interactions effectively, making them potent agents in immune response regulation, mitosis, apoptosis, and signal transduction [72]. Moreover, the complexity and multiplicity of chemical components in plant extracts make it difficult for bacteria to develop resistance compared to single-compound synthetic drugs [73,74].

## 6. Combating Resistance Through Synergism Among Phytoconstituents

Traditionally, research on medicinal plants has focused on isolating single active compounds responsible for therapeutic effects. However, this approach can lead to a reduction in the efficacy of the extract, as isolation may lose the synergy between different constituents. The enhanced activity of plant extracts is often due to the complex interplay of secondary metabolites, which may function in defense and cell signaling, thereby increasing the overall biological activity of the plant [75]. The combined action of various compounds within a single extract targets multiple sites within pathogens, including receptors, enzymes, ion channels, and transport proteins. This multitargeted approach slows the development of bacterial resistance compared to treatments involving a single active compound.

Combining plant compounds with conventional antibiotics can enhance the latter’s effectiveness and reduce side effects. Understanding the synergistic interactions between various phytochemicals can further promote the use of medicinal plants, whether used alone, in combination with each other, or alongside antibiotics. For instance, Shibata et al. (2005) found that ethyl gallate increased the susceptibility of *Staphylococcus aureus* strains to β-lactam antibiotics [76]. This synergy was specific to β-lactams and did not affect other antibiotic classes tested.

Another study highlighted the synergistic effect of 5-methoxyhydnocarpin, a compound from chaulmoogra oil, which enhances the antibacterial activity of berberine against *Staphylococcus aureus* [77]. Although 5-methoxyhydnocarpin itself lacked antimicrobial activity, it significantly increased berberine accumulation within bacterial cells by inhibiting the bacteria’s multidrug resistance pumps, illustrating how weak antimicrobial agents can be combined with other compounds to potentiate their activity.

## 7. Combining Traditional and Modern Medicine—Bioenhancers

In the face of growing antibiotic resistance, the development of new, effective antibiotics has become increasingly challenging. Current global drug development efforts may not yield new antibiotics within the next decade, despite advances in pharmacology and chemistry aimed at modifying existing antibiotics or identifying new enzyme targets [78]. Given the rising resistance to conventional antibiotics, it is logical to explore combination therapies that pair standard antibiotics with plant extracts known for their bioenhancing properties, aiming to achieve bactericidal synergy. Such combination therapies could offer novel treatment options for infectious diseases by expanding the antimicrobial spectrum, preventing the emergence of resistant strains, and reducing toxicity [79] (Table 3).

**Figure 5:**
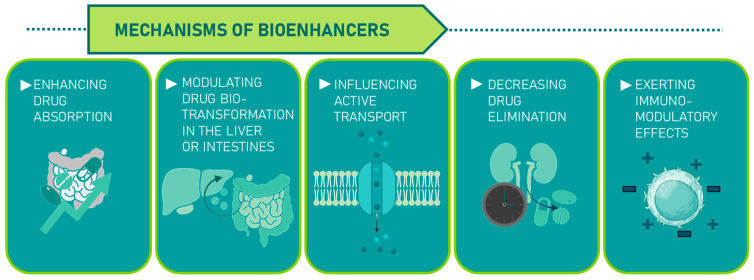
Plant-derived bioenhancers and their mechanisms.

Bioenhancers can function through various mechanisms: enhancing drug absorption, modulating drug biotransformation in the liver or intestines, influencing active transport, decreasing drug elimination, or exerting immunomodulatory effects (Figure 5) [58].

Traditional Chinese Medicine (TCM) is one of the oldest and most popular practices to this day, which is based on the use of bioactive compounds of plant origin, including in particular extracts containing numerous bio-compounds classified as plant secondary metabolites bioenhancers. Nowadays, the increasing popularity of TCM raises significant concerns regarding its safety, regulation, efficacy, and mode of action. In some instances, the use of TCM has been associated with serious adverse effects, including nephropathy and hepatitis [84,85]. Variations in the chemical composition of different brands of the same herb, often due to inadequate processing or adulteration with cheaper substitutes, have been linked to cases of poisoning. Additionally, contamination with heavy metals further exacerbates safety concerns.

TCM is deeply rooted in medical theory, and discarding this traditional knowledge while using the drugs can lead to severe consequences. It is important for individuals using dietary supplements, including Chinese herbal medicines, to have a deeper understanding of the underlying medical theories and the rationale for their use. A notable example is the misuse of the ancient formula xiao-chai-hu-tang in Japan, originally intended for treating febrile diseases in the Shaoyang meridian (imbalances in the body’s energy pathways) but later widely prescribed for long-term hepatitis treatment. This misuse led to severe adverse effects, including fatalities [84,85].

The historical success of TCM suggests its potential to broaden healthcare options, whether through single-chemical entities or complex botanical drugs. As the global population ages and chronic and degenerative diseases become more prevalent, TCM and complementary and alternative medicine (CAM) are likely to gain further acceptance. With advances in systems biology, pharmacogenomics, synergistic medicine, and personalized medicine, there is hope for the eventual integration of TCM and Western medicine, combining the strengths of both traditions to benefit patients.

## 8. Saponins

**Figure 6:**
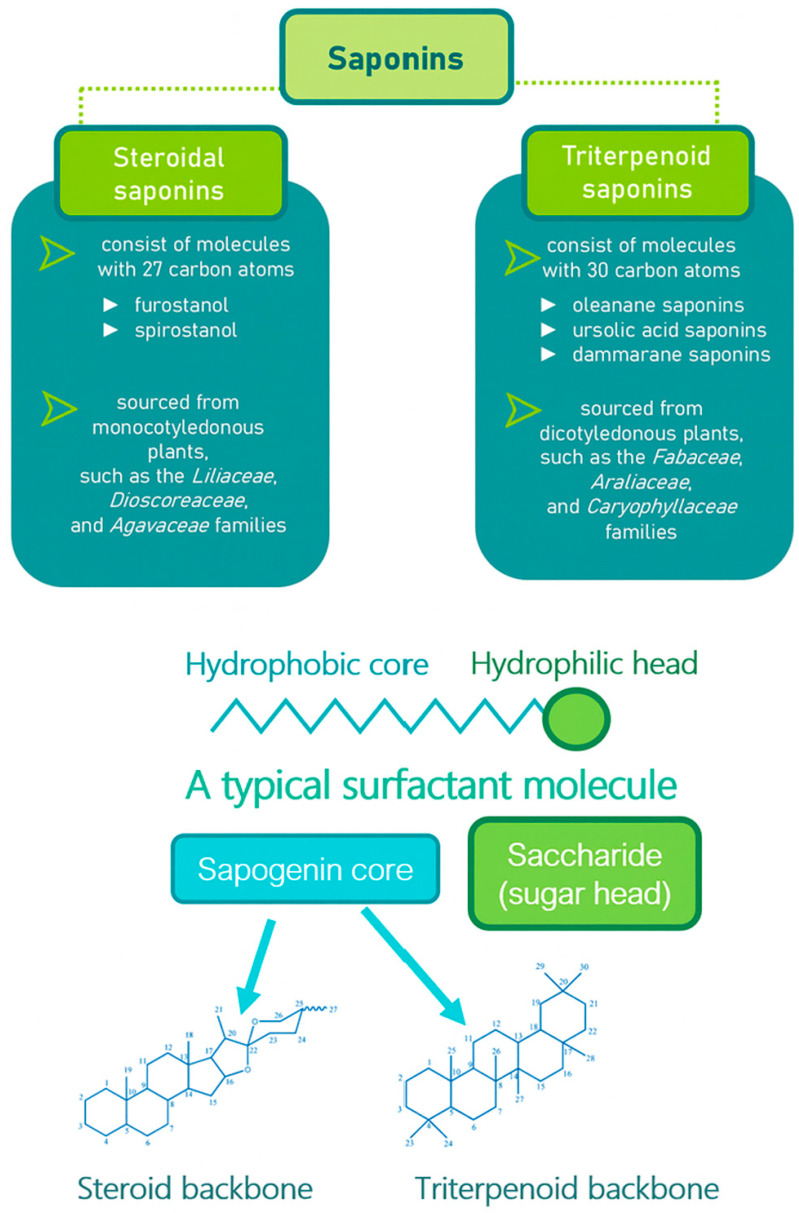
Classification of saponins by aglycone structure.

**Figure 7:**
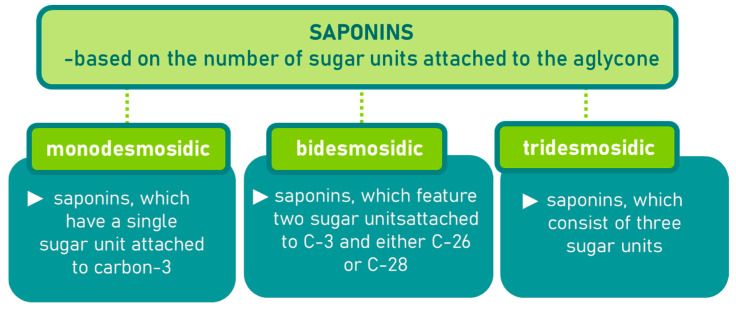
Classification of saponins by sugar unit attachment.

Saponins are a class of secondary metabolites produced by certain plants, some insects and marine organisms. The term “saponin” originates from the Latin word “sapo,” meaning soap, which reflects their ability to form a stable, soap-like foam when an aqueous solution of saponins is vigorously shaken [86]. Saponins can be extracted from various parts of a plant, including roots, leaves, fruits, pericarps, flowers, and seeds. The composition and concentration of saponins can vary not only between different plants but also among different parts of the same plant [87,88]. These variations in saponin content are influenced by environmental factors during the plant’s development, as well as the methods used for extraction. Saponins are naturally occurring amphiphilic glycosides, characterized by their structure, which includes polar glycone components (sugar moieties) and nonpolar aglycone components (sapogenins) [87,88]. These compounds are categorized based on the type of aglycone they contain into two main groups: steroidal saponins and triterpenoid saponins as shown in Figure 6. Saponins are also categorized based on the number of sugar units attached to the aglycone (Figure 7). The sugar chains attached to the aglycone can be either branched or linear, typically comprising D-glucose, D-galactose, L-arabinose, L-rhamnose, D-xylose, D-fucose, and glucuronic acid [89].

**Figure 8:**
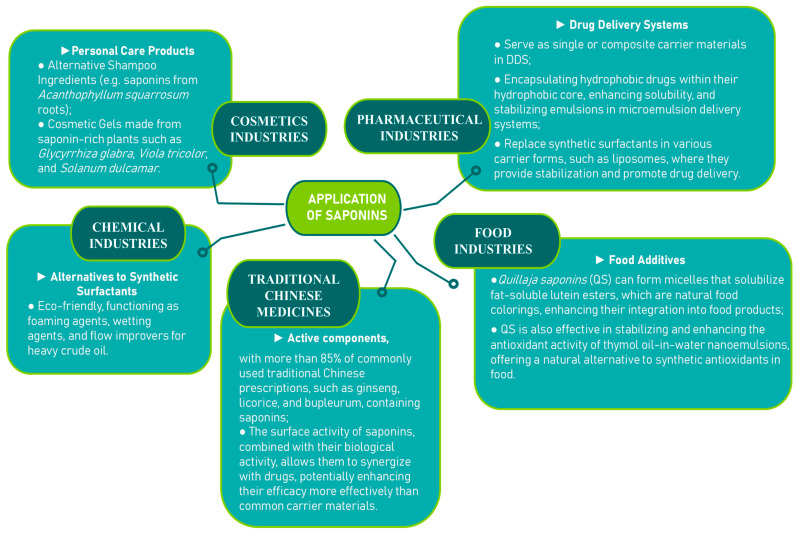
Application of saponins [90,91,92,93,94,95,96].

Due to the combination of lipophilic aglycones and hydrophilic sugar chains, saponins function as natural surfactants, making them potential substitutes for chemical surfactants. Beyond this, saponins have a wide range of applications, including in the pharmaceutical, food, and cosmetic industries [87] (Figure 8).

They exhibit numerous bioactivities, such as expectorant, anti-inflammatory, vasoprotective, hypocholesterolemic, immunomodulatory, hypoglycemic, antifungal, and antiparasitic effects [97,98,99,100,101,102] (Table 4).

Plants rich in saponins, like *Panax ginseng* and *Glycyrrhiza glabra*, have been used medicinally for centuries, particularly in Asian cultures [102]. In modern times, saponins are also employed as adjuvants in vaccine production [114,115,116]. In addition, steroidal sapogenins derived from saponins are important raw materials for the pharmaceutical industry, especially in the synthesis of steroidal hormones (Suresh et al., 2021 [117]). Recent research has highlighted the role of saponins as chemopreventive and antitumor agents across various models. Notable examples include the chemopreventive effects of ginsenosides and diosgenins, as well as the anticancer properties of saikosaponins and glycyrrhizins [118,119,120,121]. Saponins inhibit tumor growth through various mechanisms, such as inducing apoptosis and autophagy, causing mitotic arrest, reducing nitric oxide (NO) production, suppressing MMP-2 and MMP-9, and activating caspase 2 [122,123,124]. Among saponins, cycloartanes, dammaranes, oleananes, spirostanes, and furostanes have been identified as particularly potent anticancer agents [125]. Saponins serve as well with their multiple functions within Drug Delivery Systems (DDS), including traditional roles like enhancing bioavailability, minimizing side effects, enabling controlled release, and improving targeting [126,127,128]. Moreover, due to their inherent biological activity, saponins can act synergistically with drugs [99,127,129,130] (Table 5).

In summary, saponins are promising natural surfactants in DDS, offering benefits such as improved bioavailability, reduced side effects, enhanced targeting, and synergistic effects with drugs, particularly in cancer therapy. Their ability to replace traditional carrier materials like cholesterol further highlights their versatility and potential in pharmaceutical applications.

## 9. Challenges in Bioavailability of Saponins

**Figure 9:**
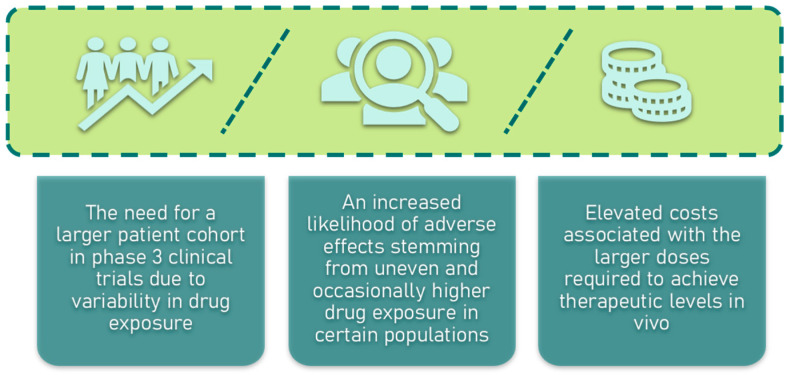
Challenges in drug development of low oral bioavailability of saponins.

One of the primary obstacles in the development of saponins as therapeutic agents is their low bioavailability. Despite the identification of over 20,000 naturally occurring saponins and more than 4000 sapogenins, their clinical potential remains limited [145]. A recent analysis highlights a trend of more than 500 new saponins being discovered annually [146,147,148]. The first report of saponins exhibiting antitumor activity dates back to 1960, involving an extract from sea cucumber [149]. Since then, around 500 studies have explored the chemopreventive or anticancer properties of saponins. However, the majority of these investigations were conducted in vitro, with only about 10% involving in vivo models, typically in mice. The poor bioavailability of saponins has often precluded their progression to human clinical trials, particularly as oral agents [150,151,152,153]. For instance, ginsenoside Rh2, a promising chemopreventive compound, has shown bioavailability rates ranging from 1% to 24.8% in animal models such as mice and dogs [153,154]. Nonetheless, in China, ginsenosides have been successfully utilized as adjuvant therapies, with some even gaining approval as pharmaceutical drugs. The low oral bioavailability of saponins presents significant challenges in drug development, particularly for human clinical trials (Figure 9) [155]. Therefore, enhancing the bioavailability of saponins is crucial, as pharmaceutical companies typically refrain from developing drugs with oral bioavailability below 30%. Given this, our review delves into the mechanisms underlying the absorption and distribution of saponins both in vitro and in vivo.

It is noteworthy that various sapogenins, which are saponin precursors, have demonstrated better bioavailability and bioactivity than their saponin counterparts [156]. This improvement is due to more favorable chemical properties, such as the absence of sugar chains. However, the relatively poor solubility of sapogenins compared to saponins is a limiting factor that also impedes a significant increase in bioavailability. Despite these challenges, there remains a scarcity of comprehensive data on the digestion, bioaccessibility, and bioavailability of saponins and sapogenins. Given the chemical diversity and complexity of saponins, it is difficult to generalize their bioavailability profiles. This underscores the need for further research to establish safe and effective oral dosing regimens.

In addition to the role of gut microbiota in modulating the bioactivity of dietary saponins, it is important to consider the impact of saponins on the microbiota itself and the subsequent health outcomes. Although current data on the relationship between saponins, microbiota, and health are limited, emerging research suggests that saponins may act as “prebiotic-like” compounds, similar to polyphenols [156]. This hypothesis is supported by recent findings, and a patent “Use of herbal saponins to regulate gut microflora” has even been filed for the use of herbal saponins to regulate gut microflora [157]. The invention proposes that plant-based saponins can promote anticancer and anti-inflammatory effects by balancing gut microbiota and maintaining a healthy epithelial environment in the gut.

The bioavailability of saponins is primarily influenced by two factors: the physicochemical properties of the saponin itself (such as molecular weight, hydrogen bond donors and acceptors, solubility, and chemical stability) and the biological barriers that hinder its absorption into the systemic circulation. These barriers include drug transport (efflux and uptake), metabolism by gut microflora, and first-pass metabolism in the intestine and liver; strategies to enhance the bioavailability of saponins are presented in Table 6.

## 10. Summary

The integration of saponins as bio-enhancers in antibiotic therapies represents a significant advance in the field of drug delivery, particularly within the context of the ongoing debate over the use of herbal excipients from complementary and alternative medicine (CAM). This scientific review has explored the multifaceted roles of saponins, particularly their ability to enhance the efficacy of antibiotics, and has addressed the challenges and controversies surrounding their use.

Saponins, as natural surfactants, have demonstrated considerable potential in enhancing the bioavailability and potency of antibiotics. Their amphiphilic nature allows them to interact with cellular membranes, potentially improving drug penetration and reducing the necessary dosage of antibiotics. This interaction is crucial in the context of antibiotic resistance, where saponins could provide a complementary mechanism to restore or enhance the activity of existing drugs. Moreover, their ability to disrupt bacterial membranes offers a promising approach to combat persistent and dormant infections, which are often resistant to conventional treatments. By enabling lower antibiotic doses, such combinatory therapies may also reduce pharmaceutical residues in the environment, contributing to the mitigation of selective pressure that drives the evolution of antimicrobial resistance (AMR) in natural ecosystems.

However, the application of saponins is not without its challenges. One of the primary concerns is their hemolytic toxicity, which raises significant safety issues when considering their use as drug carriers. The hemolytic activity of saponins, particularly at higher concentrations, necessitates careful dose optimization and possibly the development of less toxic alternatives.

Another critical challenge is the variability in saponin solubilization properties, which can either enhance or inhibit the solubility of different drugs. This variability underscores the need for a tailored approach to saponin selection and formulation, ensuring that the chosen saponin not only enhances the target drug’s bioavailability but also maintains consistent and predictable pharmacokinetic properties.

Furthermore, the practical application of saponins in drug delivery systems (DDS) remains largely at the experimental stage. The high cost of extraction and purification, along with the sustainability concerns due to the plant-based origin of most saponins, limits their widespread adoption. The example of the *Quillaja* tree, where only a small fraction of the plant is utilized, highlights the inefficiency and environmental impact of current extraction methods. Developing environmentally sustainable sourcing and processing strategies, including cultivation of medicinal plants and green extraction technologies, will be essential to ensure the ecological viability of these excipients in future pharmaceutical systems.

Despite these obstacles, the potential benefits of combining saponins with antibiotics are significant. The ability of saponins to act as bio-enhancers aligns with the broader trend of integrating CAM with modern pharmacotherapy, offering a complementary strategy to traditional antibiotic treatments. This approach not only has the potential to improve drug efficacy but also to reduce the development of antibiotic resistance by lowering the required dosage and enhancing the drug’s mechanism of action. Importantly, such strategies may also align with global efforts to reduce the environmental burden of pharmaceuticals and limit the spread of resistance-conferring genes across microbial communities.

## 11. Conclusions

The promise of saponins as antibiotic bioenhancers is clear, yet realizing this potential in clinical practice will require close attention to safety, manufacturing variability, and application pathways. The ongoing research into less-toxic saponin variants, improved extraction methods, and optimized drug formulations will be crucial in overcoming these challenges. Given their dual potential to enhance human therapeutic outcomes and reduce environmental risk factors associated with antibiotic misuse, saponins represent a promising and sustainable innovation in future antimicrobial strategies. As the field progresses, the combination of saponins with antibiotics could become a powerful tool in the fight against resistant infections, bridging the gap between CAM and conventional medicine and paving the way for more effective and sustainable therapeutic strategies.

## Figures and Tables

**Table 1:** Challenges and opportunities for membrane-active agents.

Challenge/ Opportunity	Mechanism	Example(s), Metrics or Agents *	Outcome/Impact	References
**Selective Toxicity**	Establishing structure–activity relationships (SARs) to design selective agents	Antimicrobial peptides, peptidomimetics	Guides the development of species-specific molecules that target pathogens without harming host cells	[58]
**Screening** **and Selectivity**	Counter-selective screening with bacterial membrane-damage assays	Red blood cell hemolysis assays for exclusion	Identifies bacterial-selective compounds by screening against mammalian and bacterial cells	[59]
**Physicochemical Properties** **and Delivery**	Use of specialized formulations to enable safe and effective dosing	Amphotericin B in a liposomal formulation	Allows use of higher doses, reduces systemic toxicity in fungal infections	[60,61]
			Optimizes physicochemical properties for oral and parenteral administration	
**Bactericidal** **Kinetics** **and Spectrum**	Determining bactericidal kinetics (concentration- vs. time-dependent)	Varies by agent	Allows tailored dosing regimens that minimize resistance and maximize efficacy	[62]
	Defining the spectrum of activity early in discovery	Targeting dormant bacterial infections	Guides chemical optimization and clinical development to treat persistent infections	
**Membrane Penetration Barriers**	Designing amphipathic agents for enhanced penetration of lipid-rich barriers	*Mycobacterium tuberculosis*, a Gram-negative bacterium	Overcomes cell wall barriers for effective membrane targeting	[63]
**Synergy with Host Immune System**	Testing compounds in vivo to assess synergy with the immune response	High minimum inhibitory concentration (MIC) agents in animal models	Enables identification of compounds effective in vivo despite high MIC	[5,64,65]
**Alternative** **Potency Metrics**	Developing metrics beyond MIC to assess efficacy against dormant cells	Minimum Stationary-cidal Concentration (MSC), Minimum Dormicidal Concentration (MDC)	Provides a more accurate measure of potency against non-growing bacteria	[5,25,31,66]
**Immobilization for Medical** **Devices**	Immobilizing agents on medical device surfaces to prevent contamination	Chitosan, Ceragenins	Prevents biofilm formation, reduces bacterial contamination on devices	[67]

* In rows that address methodological needs (e.g., potency readouts against non-multiplying cells), the ‘Examples’ column lists metrics (e.g., MSC, MDC) in rows that address chemistry-based opportunities, it lists compound agents.

**Table 2:** Examples of plant secondary metabolites and their antimicrobial action.

Plant Species	Secondary Metabolites	Target Microorganisms	Mechanism of Action	References
***Salvia officinalis*** ** (sage)**	Flavonoids, phenolic acids	Gram-negative and Gram-positive bacteria	Exhibits bacteriostatic and bactericidal effects in both aqueous and alcoholic extracts	[68]
***Salvadora persica*** ** L. (miswak)**	Tannis, Alkaloids	*Streptococcus*, *Staphylococcus aureus*	Shows antimicrobial activity tested via micro-well dilution and disc diffusion methods against multiple species	[69]
***Mentha piperita*** ** (mint)**	Phenolics, flavonoids	*Bacillus subtilis*	Moderate antimicrobial activity through inhibition of microbial enzymes and proteins	[70]
***Eugenia caryophyllata*** ** (cloves)**	Phenolics, Flavonoids, Terpenoids	*Bacillus subtilis*	Highest antimicrobial activity among tested plants, possibly due to polyphenolic interactions with microbial cells	[70]
***Prunus avium*** ** (cherry)**	Polyphenolic compounds	*Bacillus subtilis*	Antimicrobial action likely through oxidized compounds and nonspecific cellular interactions	[70]
***Rosmarinus officinalis*** ** (rosemary)**	Polyphenolic compounds	*Bacillus subtilis*	Inhibits microbial growth via oxidative interactions with cell structures and enzymes	[70]
***Peganum harmala***	Alkaloids	13 multidrug-resistant Gram-positive and Gram-negative strains	Antibacterial activity by enzyme inhibition, cell division disruption, and membrane alteration	[71]
***Catharanthus roseus***	Alkaloids (over 130 compounds)	*Escherichia coli*, *Candida albicans*, *Staphylococcus aureus*	Inhibits enzymes, disrupts cell division, affects respiration, and alters bacterial membranes	[71]

**Table 3:** Mechanisms of bioenhancer-mediated synergy with antibiotics.

Plant Source	Active Compound(s)	Antibiotic	Microorganism(s)	Observed Effect	References
***Rosa canina***	Tellimagrandin I 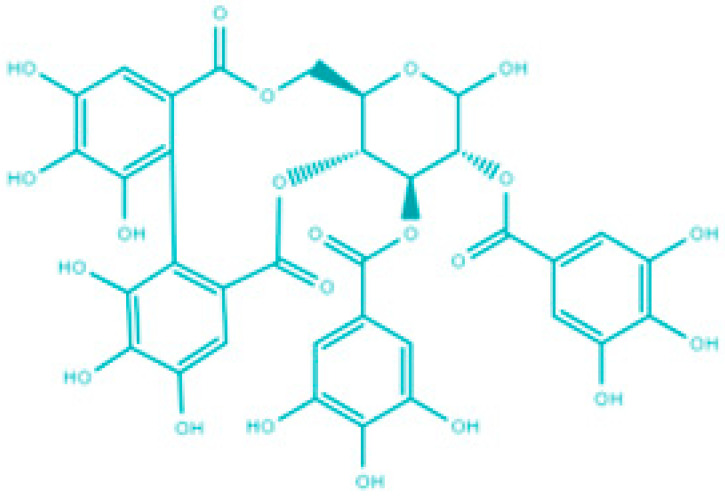	β-lactams (penicillin) 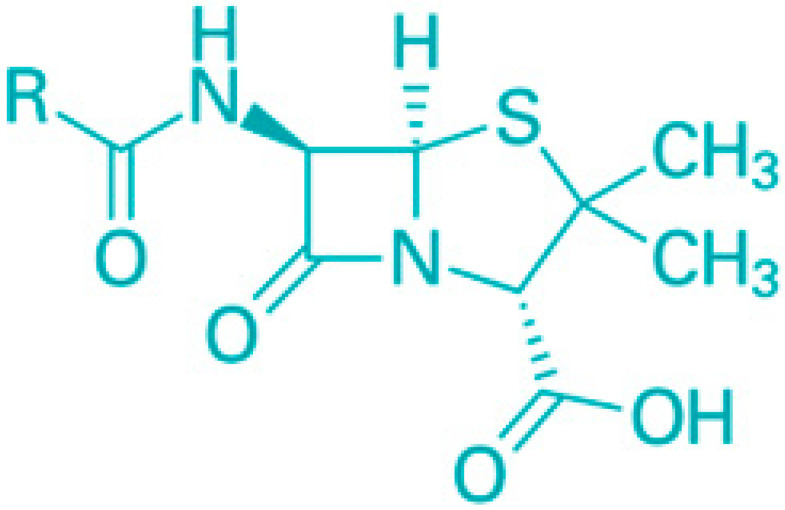	methicillin-resistant *Staphylococcus* *aureus*	Enhances the bioavailability and inhibitory effects of antibiotics	[80]
***Arctostaphylos*** ** uva-ursi**	Corilagin 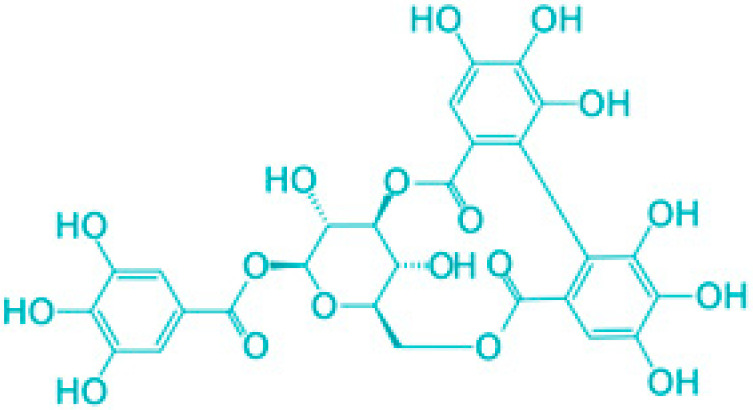	β-lactams (penicillin) 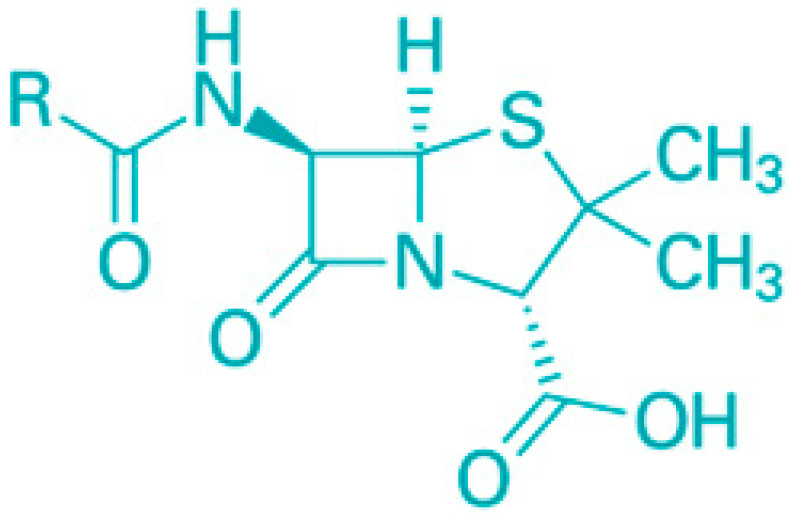	methicillin-resistant *Staphylococcus* *aureus*	Boosts the bioavailability and inhibitory effects of antibiotics	[80]
***Mangifera indica***	Mangiferin 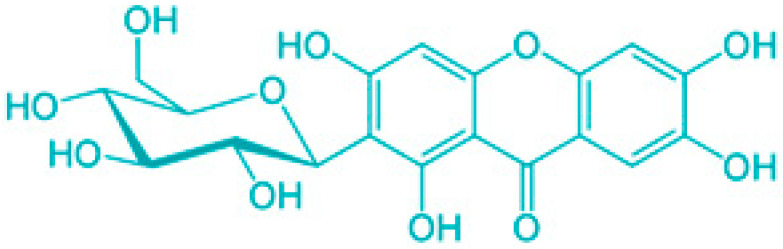	Tetracycline, 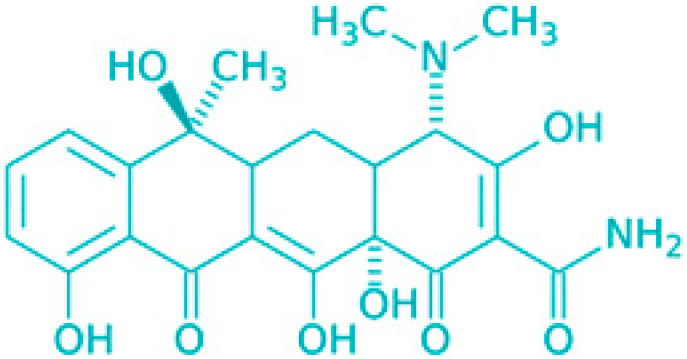 Erythromycin 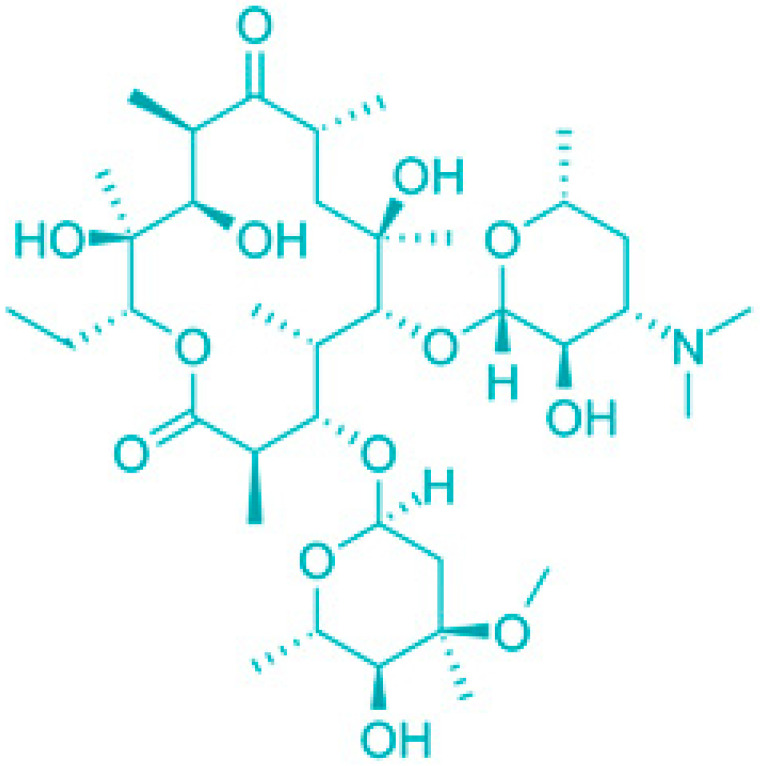	*Staphylococcus aureus*	Fourfold reduction in MIC * when combined with tetracycline and erythromycin	[81]
***Tectona grandis***	Tectoquinone 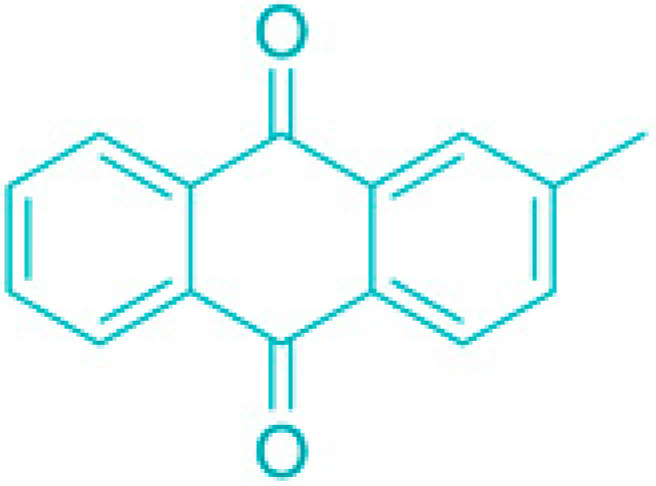	Tetracycline 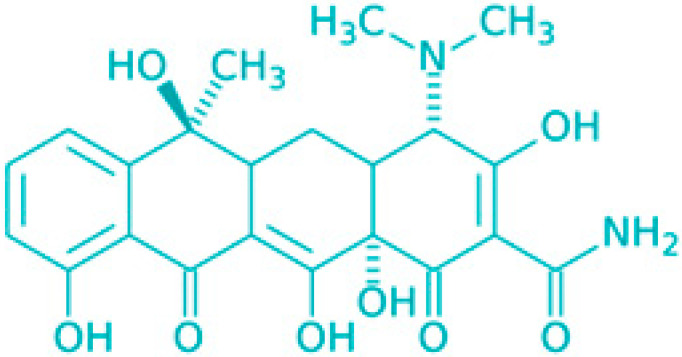	*Salmonella typhimurium*, *Klebsiella pneumoniae*	Twofold MIC * reduction for *Salmonella typhimurium* and fourfold MIC * reduction for *Klebsiella pneumoniae*	[82]
***Pseudolarix kaempferi***	Pseudolaric acid A 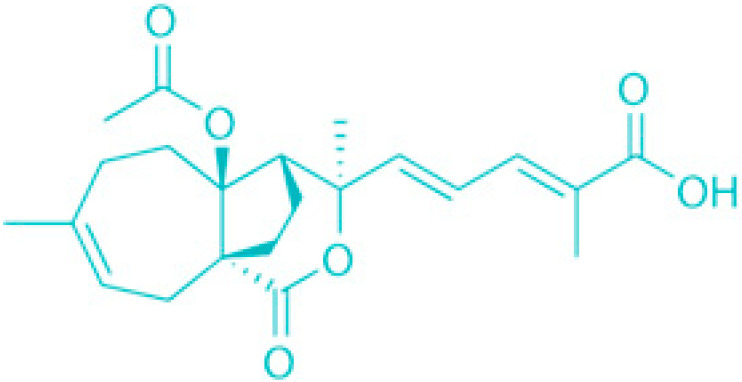	Fluconazole 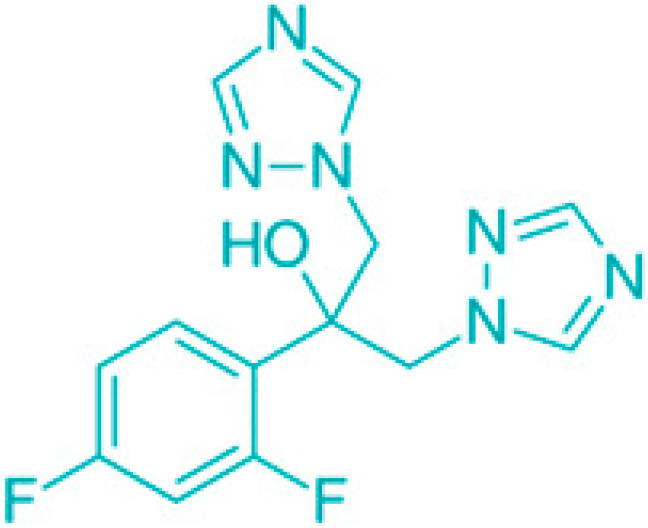	*Candida* species	Enhanced antifungal effectiveness against *Candida* species	[83]

* MIC—minimum inhibitory concentration.

**Table 4:** The bioactivity of saponins.

Biological Activity	Description	Saponin Source	Targeted Pathogens or Effects	Key Findings	References
Antimicrobial	Broad-spectrum antibacterial and antifungal activity by damaging cell walls and membranes	*Chenopodium quinoa* (quinoa)	*Staphylococcus aureus*, *Staphylococcus epidermidis*, *Bacillus cereus*	Severe bacterial cell damage, including cell wall degradation and membrane disruption	[103]
		*Ziziphus joazeiro*	*Candida albicans*, *Aspergillus niger*	Antifungal action with similar membrane disruption	[104]
Antioxidant	Reduces oxidative stress by neutralizing free radicals	Camellia roots and seed cakes	Oxidative damage	Notable antioxidant potential	[104]
		*Agave sisalana* (steroidal)	-	Similar antioxidant activity demonstrated by saponins	
Antidiabetic	Regulates blood glucose and lipid levels	*Panax notoginseng*	Blood glucose levels	Reduces elevated blood glucose in diabetic models	[105,106,107]
		*Entada phaseoloides* seeds	-	Similar glucose-lowering effects observed	
		*Stauntonia chinensis*	Blood glucose and lipid levels	Hypoglycemic and hypolipidemic activities in diabetic mice	[108]
Immunomodulatory	Enhances immune response and serves as an immunological adjuvant	*Chenopodium quinoa* seeds	Immune response	Enhances immune response and hemolytic activity in mouse models	[108,109]
		*Bupleurum chinense* roots	-	Similar immunomodulatory effects	
		*Quillaja*, *Glycine max* (soya), Japanese ginseng	Immune system	Demonstrates adjuvant properties, enhancing immune response	[110,111,112]
Hemolytic Activity	Lyses red blood cells in vitro, varying by saponin source	*Silene vulgaris*	Hemolysis	Moderate hemolytic activity, less potent than other saponin sources	
		*Sapindus mukorossi, Chlorophytum borivilianum*	-	High hemolytic activity compared to *Silene vulgaris*	[113]

**Table 5:** Functions of saponins in Drug Delivery Systems (DDS).

Function	Mechanism	Example	Effect/Outcome	References
**Enhancing Bioavailability**	Increasing Drug Solubility	- *Quillaja* saponins (QS) and cholesterol	- 103× increase in cholesterol solubility	[131]
		- Curcumin encapsulation	- Increased bioavailability of curcumin by 8.9–19×	
		Vitamin K, Atorvastatin Calcium, Prazquantel	Improved solubility through encapsulation in micelles	[132,133,134,135,136]
	Enhancing Membrane Permeability	α-Hederin, Glycyrrhizic Acid (GA)	- α-Hederin forms transient pores in membranes	[136]
			- GA increases praziquantel permeability	
		Ginsenosides with ciprofloxacin	Inhibits P-glycoprotein (P-gp), promoting intestinal drug permeability	[137]
		GA as a transdermal enhancer	Disrupts lipid bilayer for improved drug absorption	[138]
**Reducing Side Effects**	Decreasing Drug Toxicity	GA with non-steroidal anti-inflammatory drugs	Reduces gastric mucosa irritation, less ulceration	[139]
		GA with fluoxetine	“Fluoglyzin” complex increases the median lethal dose (LD50), lowers the dose needed	[140]
**Targeted Drug Delivery**	Liver Targeting	GA receptors on liver cells	Enhanced uptake in HepG2 hepatocarcinoma cells	[141]
		GA and curcumin composite gel	Targeted delivery to liver, improved cell uptake	[142]
**Synergistic Effects**	Anticancer Activity	GA, ginsenosides, diammonium glycyrrhizinate	Inhibits multiple drug resistance, reduces drug efflux in tumor cells, enhancing effect	[137]
		Saponin complexes with paclitaxel	Enhanced anticancer effects, improved circulation	[143]
**Alternative Carrier Potential**	Cholesterol Replacement in Liposomes	Ginsenoside Rh2 and Rg3	Maintains particle size, improves encapsulation efficiency	
		Timosaponin AIII	Alternative to cholesterol, better circulation without oxidation risk	[144]

**Table 6:** Strategies to enhance bioavailability.

Strategies to Enhance Bioavailability	Approach	Mechanism	Examples
**Synthesis of Derivatives and Nanoparticle Formulations**	Modify molecular structure to enhance solubility, or use nanoparticle-based delivery.	Increases aqueous solubility and bioavailability by altering chemical properties or using nanoscale carriers.	Amino acid derivatives of oleanolic acid, liposomes, microemulsions, nanosuspensions, nanocapsules [158].
**Use of Antibiotics to Inhibit Microflora Degradation**	Antibiotics prevent hydrolysis of saponins by gut microflora.	Reduces the breakdown of saponins by gut bacteria, potentially improving systemic bioavailability.	Pretreatment with amoxicillin or metronidazole improves glycyrrhizin bioavailability [159,160].
	**Limitations:** Long-term antibiotic use can disrupt gut microbiome, cause adverse effects, and is not viable for chemopreventive applications.		

## Data Availability

No new data were created or analyzed in this study, data sharing is not applicable.

## Abbreviations

The following abbreviations are used in this manuscript:

TB	Tuberculosis
HIV	Human Immunodeficiency Virus
ATP	Adenosine Triphosphate
US CDC	United States Centers for Disease Control
OM	Outer Membrane
OmpA	Outer membrane protein A
OmpF	Outer membrane protein F
OmpT	Outer membrane protein T
LPS	Lipopolysacharides
Lpp	Lipoprotein
PMBN	Polymyxin B Nonapeptide
MMP-2	Metalloproteinase 2
MMP-9	Metalloproteinase 9
TCM	Traditional Chinese Medicine
CAM	Complementary and Alternative Medicine
DDS	Drug Delivery System
GA	Glycyrhhizic Acid
PSMs	Plant Secondary Metabolites
MIC	Minimum Inhibitory Concentration
LD50	Median lethal dose/lethal dose, 50%
QS	*Quillaja* Saponins
